# The prophet’s rite of passage – pitfalls in evaluating real-time prediction in medicine

**DOI:** 10.3389/fphys.2025.1569008

**Published:** 2025-04-09

**Authors:** Noam Keidar, Yael Yaniv

**Affiliations:** Laboratory of Bioelectric and Bioenergetic Systems, Faculty of Biomedical Engineering, Technion-IIT, Haifa, Israel

**Keywords:** prediction, atrial fibrillation, epilepsy, clinic, real-time prediction model

## Introduction

The future has always captivated human imagination, with efforts to assess disease prognosis dating back to ancient Egyptian times: “*If the heart trembles, has little power and sinks, the disease is advancing … and death is near … ”* (Papyrus Ebers, circa 1550 BC). However, the risks of relying on predictions were also acknowledged in antiquity: “…The prophecy has been taken from the prophets and given to the fools and babies instead … ” (Babylonian Talmud: Baba Bathra 12b). Recent advancements in medicine have significantly enhanced prognostic accuracy. The availability of comfortable and reliable wearable devices capable of measuring cardiac, neuronal, and other physiological signals, combined with sophisticated machine learning algorithms designed to interpret these signals in real time (Davoodi et al., 2024; Elul et al., 2024; Fira et al., 2024; Kerr et al., 2024), marks the dawn of a new era in preventive medicine—the age of real-time prediction.

### Prediction vs. risk factor

Scanning the existing literature reveals considerable semantic confusion surrounding the terms prediction, risk factor identification, detection, and diagnosis, which are often used interchangeably. To clarify, we propose the following distinctions:

#### Prediction

Definition: “Event X is likely to occur within time interval T for individual P.”

Relation: Future-oriented; depends on statistical/machine learning models and longitudinal data.

Example: “This patient has a 30% chance of developing heart failure within the next 5 years.”

#### Risk factor

Definition: “Individual P is at higher risk for event X due to factor F.”

Relation: Identifies modifiable/non-modifiable contributors to an event.

Example: “Hypertension is a risk factor for stroke.”

#### Detection

Definition: “Individual P is currently experiencing event X.”

Relation: Real-time or near real-time identification of an ongoing event.

Example: “A wearable ECG detects atrial fibrillation in real time.”

#### Diagnosis

Definition: “Individual P has condition D.”

Relation: Typically involves clinical assessment, imaging, or lab tests.

Example: “The patient has been diagnosed with myocardial infarction based on ECG and biomarker analysis.”

In summary, we can define these conceptual relations:

Prediction → Risk Factor Identification (Prediction models often incorporate risk factors.)

Risk Factor Identification → Prediction (Identified risk factors improve predictive accuracy.)

Detection → Diagnosis (Detected anomalies may trigger diagnostic confirmation.)

Diagnosis → Prediction (Diagnosis may inform predictions about disease progression.)

### New era of prediction

Traditionally, preventive medicine has focused on risk factor identification and mitigation, screening tests, patient and caregiver education, and the deployment of response systems for life-threatening events. However, the advent of real-time prediction is transforming this discipline. Imagine a world where non-invasive wearable devices issue alerts before the onset of various conditions, such as life-threatening cardiac arrhythmias, heart failure decompensation, or stroke. These capabilities could pave the way for precisely timed, real preventive therapies, enabling clinicians to achieve optimal outcomes with fewer interventions while preventing serious medical events and their potentially life-threatening consequences.

Such real-time prediction systems are already being developed by research groups and companies, showing promising results (Fira et al., 2024; Kerr et al., 2024). Yet, the true clinical value and significance of these advancements can be challenging to define and fully comprehend.

### Alarm goes off

The essence of a real-time prediction is the alarm, an actionable output presented to patients and/or caregivers at the right time, to convey a clear message and facilitate the most appropriate intervention. If the alarm does not warrant a specific action, not accurately timed or has an ambiguous meaning, its clinical utility might be questionable. While these requirements may seem obvious, the usual tools used for evaluation of prediction systems are limited due to unique challenges posed by the continuity of time. To validate that a prediction system has a positive effect on human health, the clinical setting, the intended use, and the continuum of time must be considered, as demonstrated in the following examples.

Unlike a classic predictive test that can be either “positive” or “negative” but not both, an alarm at a certain time may be both or neither. Imagine a system predicting sudden cardiac arrests and activating Emergency Medical Services (EMS), enabling immediate intervention on onset. An alarm 20 s before the event is useful, but considering response times of even the best EMS, no added benefit is gained. Such alerts are indeed true positive as the event was accurately predicted, but false negative in the sense that it does not support a timely response. Similarly, an alarm set off 5 min after onset is technically false, but not “as false” as an alarm calling the EMS for no reason at all.

Another challenge when testing a prediction system is consideration of the distribution of false or true alarms over time. For example, 30 false alarms within the same hour will mean a single unnecessary EMS activation, but when issued every few days over 4 months, will repeatedly unnecessarily activate EMS, and result in patient anxiety and stress as well as complacency, possibly leading to missed true alarms. On the other hand, even a single timed alarm is enough for intervention, making any “false negative” that relates to the same event meaningless.

### How to report real time prediction results?

To illustrate the complexity of the problem, consider the example of predicting out-of-hospital sudden cardiac arrest. The incidence of the predicted event is approximately 55 cases per 100,000 person-years (Berdowski et al., 2010). But many studies showing event prediction results calculate sensitivity, specificity, and accuracy for patient groups with even representation for positive and negative intervals, introducing a selection bias. In our example, even if we consider an entire day before the event as a relevant prediction window, 99% specificity in a 1:1 positive-negative sample population is equivalent to 0.0003% specificity.

Researchers from different scientific fields approach this problem in various ways. Earthquake prediction research commonly uses a predictive ratio (Kagan and Knopoff, 1987) that compares the predicted probability in space and time against a null hypothesis assuming the events are random (Poisson stochastic process). This method is unbiased and rigorous, but sheds little light on the expected clinical utility if adopted to medicine. Arrythmia predictions for example, usually use samples of positive and negative intervals (e.g., the paroxysmal atrial fibrillation prediction challenge (Moody et al., 2000) uses a 1:1 positive-negative ratio), which provides clear clinical trial metrics (sensitivity, specificity, *etc.*) but introduces a selection bias. Epileptic seizure prediction studies usually show metrics on a sample of intervals, adding the false alarm rate per hour (Daoud and Bayoumi, 2019), a measure useful for estimation of false alarm burden on patients and healthcare systems, but sensitive to the alarm time-distribution problem described above.

### Authors solicited opinion regarding real-time prediction system results

We are of the opinion that introduction of real-time prediction systems to clinical practice requires a usage-centered approach. This would first require definition of the action that patients and caregivers should take once an alarm is raised, e.g., “activate EMS, then observe the patient for 6 h after the alarm”. Once the action is clear, a true positive can be appropriately defined, i.e., for each event, define if the alarm issued at a relevant time for the action is efficient. For example, assuming an 8-min notice is needed to significantly improve patient survival, all events with at least one alarm between 6 h and 8 min before onset would be considered true positive. Thereafter, sensitivity can be defined as the rate of true positives vs. events. A false positive would be a needless action, e.g., an EMS activation. As the patient is observed for 6 h, this is the minimal allowed gap between two activations; two false alarms 3 h apart would be considered a single false positive alarm. These definitions of true and false positives can then be applied to meaningfully determine the positive predictive value. Alarms not achieving the goal but not causing an irrelevant action, e.g., an alarm 20 s before or during an event, are neither positive nor negative.

## Conclusion

We hope that clear definitions, awareness of pitfalls and the proposed approach will help set a benchmark for clinically relevant real-time prediction systems, creating the prophet rite of passage, and retrieving prophecy from the fools.
